# Transitioning from lateral to the prone transpsoas approach: flatten the learning curve by knowing the nuances

**DOI:** 10.3171/2022.3.FOCVID2224

**Published:** 2022-07-01

**Authors:** Nima Alan, Jared J. Kanter, Lauren Puccio, Sharath Kumar Anand, Adam S. Kanter

**Affiliations:** 1 Department of Neurological Surgery, University of Pittsburgh Medical Center, Pittsburgh, Pennsylvania; and; 2 Department of Communications–Media, University of Alabama, Tuscaloosa, Alabama

**Keywords:** lateral lumbar interbody fusion, LLIF, prone transposes, single-position lateral, lateral access spine surgery

## Abstract

Prone transpsoas lateral lumbar interbody fusion is the newest frontier in surgical approach to the lumbar spine. Prone positioning facilitates segmental lordosis and facile posterior segmental fixation. However, even in experienced hands, transitioning from a lateral decubitus to prone position necessitates alterations to the traditional technique. In this video, the authors highlight the nuances of adopting the prone transpsoas lateral lumbar interbody fusion technique and strategies to overcome them.

The video can be found here: https://stream.cadmore.media/r10.3171/2022.3.FOCVID2224

## Transcript

This is a video presentation of nuances of adopting prone transpsoas approach to the lateral lumbar interbody fusion technique. Compared to its traditional lateral decubitus position, prone transpsoas has the benefit of achieving natural segmental lordosis, improving work efficiency, and allowing single position for both lateral and posterior access to the spine.^1–3^ However, there are nuances to the adoption of this technique, which are highlighted in this presentation.^4–8^

### 0:46 Patient Preoperative Description.

This is a 46-year-old female with BMI of 50 with longstanding history of back pain who did not get any relief with nonsurgical management. Was found to have a mobile L3–4 spondylolisthesis with an MRI that showed favorable anatomical features to undergo a lateral lumbar interbody fusion. The patient was consented for a prone transpsoas lateral lumbar interbody fusion.

### 1:09 Surgery Start.

The patient was brought to the operating room and placed in a prone position on a bumpy Jackson table and secured in position using I-band and 3-inch tape. Using fluoroscopy, we marked the L3 and L4 pedicles and the midline posteriorly, and the L3–4 disc space was also marked using a K-wire. The patient was prepped and draped in the usual fashion.

### 1:32 First Nuance: Depth of the Surgical Field.

The first nuance of prone transpsoas lateral lumbar interbody fusion is the depth of the surgical field, especially in patients with truncal obesity such as our patient with a BMI of 50. You can see that the surgeon is struggling to reach across to transverse the psoas. This nuance is overcome by the assistant surgeon providing counterpressure from the contralateral side to push the spine toward the surgeon, reducing the depth of the surgical field. We highlight here that the longest blade for retraction provided by the vendor was used to do the surgery.

### 2:05 Second Nuance: Dock Anteriorly.

The second nuance of prone positioning for transpsoas lateral lumbar interbody fusion is a tendency to dock anteriorly. This is due to the fact that one, gravity pulls all instrumentation downward, so the surgeon has to be conscious of this and persistently attempt to point posteriorly. Secondly, the prone positioning of the patient translates to the lumbar plexus posteriorly, which favors the surgeon to dock anteriorly. This is an important nuance for the surgeon to be aware of because the anterior docking could result in a potential injury to the major vessels and anterior longitudinal ligament. During this case, we were able to land at the midline of the L3–4 disc space. However, there are case reports of injury to the anterior longitudinal ligament in the literature.

### 2:50 Third Nuance: Surgeon Ergonomics.

The third nuance of prone positioning for transpsoas lateral lumbar interbody fusion is pertaining to surgeons’ ergonomics. You can see here that the surgeon, with traditional loops that are designed to look downward, has to extend his neck and hunch over to visualize the disc space. The arms have to be abducted and lifted up, which creates an unsustainable and uncomfortable position for the surgeon. A reported adjustment in the literature for this nuance is to rotate the bed 15° to 20° in order to make the surgery more ergonomic. However, this requires the x-ray to also adjust accordingly to maintain orthogonal during the surgery in order to have a parallel approach to the endplates and not violate the endplates, which would result in intraoperative subsidence. We were able to deploy the shim while remaining in a sitting position. Here we are doing the disc preparation, highlighting again that the depth of the surgical field is increased, the whole length of the Cobb has to be used, and the surgeon remains in an awkward position while sitting.

### 3:52 Fourth Nuance: Lack of Counterpressure.

The fourth nuance of prone positioning for transpsoas lateral lumbar interbody fusion is a lack of counterpressure during the surgery. This is particularly pronounced during the deployment of the interbody device, which requires malleting of the instrumentation. With each percussion, the retraction tends to move out of place and anteriorly, which could be potentially dangerous, putting the major vessels and anterior longitudinal ligament at risk. In order to overcome this nuance, the assistant surgeon holds the retraction in place during the deployment of the interbody device. During this surgery, we first were not aware of this nuance and the retraction fell out of place and we had to reinsert the K-wire and find our way back into the disc space. We were able to deploy the interbody device at L3–4 in a desirable position. We then proceeded to place the pedicle screws while the patient remained in a prone position, which is one of the advantages of prone transpsoas lateral lumbar interbody fusion with instrumentation placed while the patient remains in a prone position, which is familiar for the surgeon.

### 4:53 Fifth Nuance: Duration of Surgery and Retraction Time.

The final nuance of prone transpsoas lateral lumbar interbody fusion that requires adjustments is due to the prolonged duration of surgery and retraction time. It is well known in the literature that risk of lumbar plexus injury increases with prolonged retraction time.^9^ During this case, the retraction time was 30 minutes due to the multiple adjustments that had to be made during each step of the surgery, as outlined previously. To mitigate this risk, we recommend periodic release of retraction to decrease the likelihood of lumbar plexus injury.

### 5:29 Summary.

In summary, transition from lateral decubitus to prone transpsoas LLIF has nuances that require certain adjustments intraoperatively. Number 1 is the depth of the surgical field. We were able to overcome this by pushing the contralateral side toward the surgeon to reduce the depth of the surgical field. Number 2 is the tendency to dock anteriorly. The surgeon must be conscious of this tendency and persistently attempt to aim posteriorly to avoid anterior docking, which puts the anterior longitudinal ligament and the great vessels at risk. Number 3 is the surgeon ergonomics that is uncomfortable if the surgeon remains in a sitting position. To address this, we recommend that the surgeon rotates the table about 15° to 20° with reciprocal adjustment in the fluoroscopy machine to remain orthogonal to the endplates. Number 4 is the lack of counterpressure from the contralateral side, which is particularly pronounced during the deployment of interbody device. This tends to push the retraction out of place. In order to overcome this, the assistant surgeon must provide stability to the retraction during the deployment of the interbody device. Number 5 is the inherent learning curve associated with adopting any new surgery. This translates to increased duration of a surgery and, more importantly, increased retraction time. The surgeon must be aware of this transition and reduce retraction periodically during surgery to avoid injury to the lumbar plexus.

## Disclosures

The authors report no conflict of interest concerning the materials or methods used in this study or the findings specified in this publication.

## Author Contributions

Primary surgeon: AS Kanter. Assistant surgeon: Alan. Editing and drafting the video and abstract: Alan, JJ Kanter, Puccio, Anand. Critically revising the work: all authors. Reviewed submitted version of the work: all authors. Approved the final version of the work on behalf of all authors: Alan. Supervision: AS Kanter.

## Supplemental Information

### Patient Informed Consent

The necessary patient informed consent was obtained in this study.
